# Prostate cancer in omics era

**DOI:** 10.1186/s12935-022-02691-y

**Published:** 2022-09-05

**Authors:** Nasrin Gholami, Amin Haghparast, Iraj Alipourfard, Majid Nazari

**Affiliations:** 1 Hematology and Oncology Research Center, Tabriz University of Medical Sciences, Tabriz, Iran; 2 GenomeFan, Tehran, Iran; 3 Institutitue of Biology, Biotechnology and Environmental Protection, Faculty of Natural Sciences, University of Silesia, Katowice, Poland; 4 Department of Medical Genetics, Faculty of Medicine, Shahid Sadoughi University of Medical Sciences, Yazd, Iran; 5 P.O. Box 14155-6117, Shiraz, Iran

**Keywords:** Prostate cancer, Genomics, Proteomics, Metabolomics, Diagnosis, Computational algorithm

## Abstract

Recent advances in omics technology have prompted extraordinary attempts to define the molecular changes underlying the onset and progression of a variety of complex human diseases, including cancer. Since the advent of sequencing technology, cancer biology has become increasingly reliant on the generation and integration of data generated at these levels. The availability of multi-omic data has transformed medicine and biology by enabling integrated systems-level approaches. Multivariate signatures are expected to play a role in cancer detection, screening, patient classification, assessment of treatment response, and biomarker identification. This review reports current findings and highlights a number of studies that are both novel and groundbreaking in their application of multi Omics to prostate cancer.

## Introduction

Recent advances in omics technology have prompted unexpected attempts to define the molecular changes underlying the onset and progression of a variety of complex human diseases, including cancer [1]. Identifying accurate, early signs of disease is a primary goal of biomedical research, which has entered an unprecedented era due to technological advances. New tools now allow characterization of large biological systems in great detail and with unprecedented resolution, rather than focusing research efforts on individual chemicals, metabolic pathways, or cells of interest [2]. The goal of omics technology is to study changes in DNA, RNA, proteins, and other biological molecules of various types in different species and individuals [3]. Continued developments in sequencing technology and the incorporation of high-throughput Omics data provided invaluable data to untangle the complexity of biological systems at several dimensions. To name a few, this comprises genomics, transcriptomics, proteomics, epigenomics, and metabolomics. Omics and functional genomics investigations have begun to discover and reveal characteristic features of tumor growth, such as main drivers of oncogenic signaling, and therapy response mechanisms [4]. Cancer biology has become increasingly reliant on the generation and integration of data generated at these levels.

Prostate cancer (PCa) is the second most common malignancy and the fifth leading cause of cancer-related mortality [5]. Up to 40% of men with PCa have no clinical signs because it is generally a slow-growing tumor. However, if PCa is detected at an early stage, patients have a survival rate of more than 99%; if it is detected at an advanced and highly metastatic stage, the survival rate is only 30% [6]. Clinicopathologic indices such as tumor stage, Gleason score, and (prostate specific antigen) PSA level are currently used to assigned patients into clinical risk groups (low, intermediate, or high-risk) [7]. In the early stages of the disease, current clinicopathologic indices do not discriminate well across patients with varied survival prospects [8]. Early PCa detection is primarily based on PSA testing, which has significant limitations. This test is far from ideal, however, because even slightly elevated PSA levels can be associated with confounding factors such as benign prostatic hyperplasia (BPH) or prostatitis [9]. In addition, more than 25% of men with PCa are found to have normal PSA levels [10, 11].

**Fig. 1:**
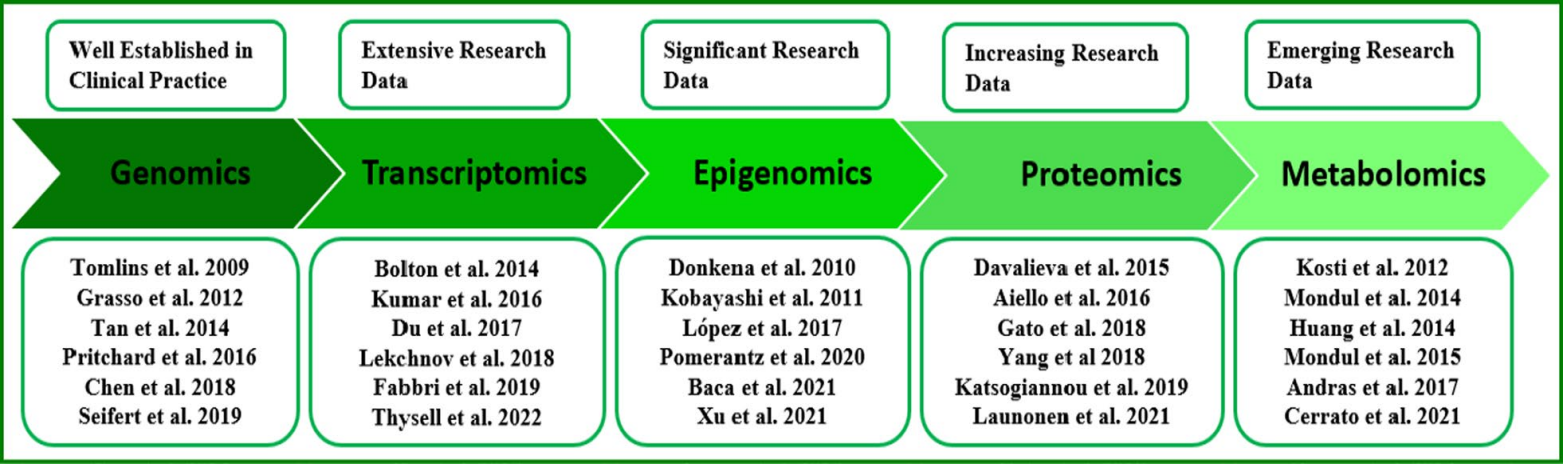
A summary of the applications of several omics technologies, as well as studies on PCa

All these facts highlight the urgent need for innovative biomarkers to improve clinical outcomes and treatment of PCa. Rapid improvements in molecular technology have enabled the identification of a number of putative PCa biomarkers. Thus, the aim of this review is to present the main findings on cancer from recently published Omics, single-cell Omics, and multi-Omic studies, as well as current computational algorithms (CAs) and cancer databases for integrating and deciphering the increasing data. The necessity of combining multi-omic data over single-omics analysis is emphasized in this review. We believe that the information provided will help readers gain a more systematic and thorough understanding of the application of joint analysis of multiple Omics data in PCa. The general picture of single Omics application in PCa is presented first, followed by a summary of research advances in the joint analysis of Omics data (Fig. 1).

## Single omics in PCa

### Genomics

A vast number of susceptibility loci have been discovered as a result of genome-wide association studies, which have highlighted the significance of genetic variations in the etiology of PCa [12]. The characterization of PCa gene drivers is the base for defining disease subgroups and developing therapeutic options of precision medicine strategies. Signature somatic gene alterations in *AR*, *PI3K-PTEN*, *WNT*, DNA repair, and cell cycle signaling pathways are found in almost all metastatic and most primary PCa patients [13]. In multiple large genomic cohort studies of primary PCa and metastatic castration-resistant prostate cancer (mCRPC), a number of DNA copy number variations (CNV), gene mutations, gene rearrangements, and gene fusions have been associated to disease recurrence [13, 14]. Furthermore, familial mutations in transcription factors such as *HOXB13* [15], tumor suppressor genes such as *TP53* and *APC* [16], and DNA repair genes such as *POLD1*, *BRCA1* and *BRCA2* [17, 18], have also been discovered in PCa genomic studies.

In addition to single nucleotide variants, other classes of driver aberrations such as gene fusions in 60%, driver CNV in 54%, and homozygous driver deletions in 50% were also very common [19]. While TP53, *PTEN* and *RB1* showed deletion peaks, *AR* and *CCND1* showed predicted recurrent CNV peaks [20]. Another study by Seifert and colleagues demonstrated that radio-resistance of the DU145 and LNCaP PCa cell lines had a direct association with CNVs using a network-based approach. Remarkably, based on driver oncogenes, it was possible to distinguish between early and late relapse groups in irradiated PCa patients [21]. Translocations of androgen-regulated promoter regions and transcription factors of the *ETS* family, such as *ERG* and *ETV*, are the most common genomic alterations in PCa [22, 23]. Chinnaiyan’s group analyzed a recurrent fusion of the 5′-UTR of *TMPRSS2* and *ERG* (TMPRSS2: ERG) as the first translocation discovered in PCa. Localized PCa has a 50% chance of TMPRSS2: ERG fusion [24, 25]. In nearly one-third of fatal mCRPC, the ETS2 gene is deleted, which is usually achieved by fusion of TMPRSS2 and ERG. Moreover, mouse intraepithelial prostatic neoplasia, a precursor lesion of prostate cancer, develops in transgenic mice producing the TMPRSS2: ERG fusion [26]. Recent genomic investigations have revealed that mCRPC with neuroendocrine symptoms frequently has *RB1* and *TP53* gene deletions, as well as a lower *AR* signal [27].

Interestingly, the incidence and prognosis of PCa vary by ethnic group, with African men having the greatest rates of incidence and mortality [28]. Later research discovered that some of the distinctions between races were attributable to genetics [29]. In a study conducted in a Chinese cohort, *FOXA1* mutations were detected in 41% compared with 4% in the Western population based on the TCGA database [30]. Moreover, the mutational spectrum of *FOXA1* in the Chinese cohort was centered on the fork-head domain, whereas in tissue samples from the Western cohort, mutations covered the entire coding sequence. Moreover, *FOXA1* mutations correlated with higher *FOXA1* expression in tumors and were associated with poor prognosis. Similarly, *ZMYM3*, *SPOP*, and *KDM6A* were also significantly mutated in the Chinese cohort compared with the Western population [30].

### Transcriptomics

Transcriptomics is the study of an organism's total number of RNA transcripts. At least 11 RNAs have been found, with mRNA, which is formed by DNA transcription and eventually translated into proteins, currently being the most interesting RNA in cancer [31]. The transcriptomics is often created as a measure of gene expression to capture the subtypes of specific tumors, as many genes are expressed similarly and are closely associated to each other [32].

Marzec et al. used a holistic method to reconstruct the molecular profile of PCa and trace the changes in mRNA levels from normal prostate to high-grade prostatic intraepithelial neoplasia and metastatic disease, providing the first full insight into its progression. They reported nine previously undiscovered stage-specific candidate genes with prognostic value. *GSTP1* and *MYC*, as well as *TP63*, *EZH2*, *CENPA*, and *PIK3CB*, were linked to tumor initiation and progression, respectively [33]. Similarly, Alkhateeb and colleagues combining several PCa gene expression datasets, have found that *DDC*, *HEATR5B*, and *GABPB1-AS1* genes show differential expression in malignant samples, suggesting that they could be used as biomarkers for PCa [34]. A valuable source of noninvasive PCa biomarkers is the urinary transcriptome. In a study by Goicoechea et al., the total urine of low-stage PCa patients (LS), high-stage (HS) PCa patients, and benign prostatic hyperplasia (BPH) patients was analyzed. *UACA* content could be distinguished between BPH and PCa patients and healthy controls. In addition, *OSBP*, *BRPF1*, and *PHC3* discriminate between LS and HS PCa patients and could be used as biomarkers to identify the early stages of the disease to guide the application of local therapy [35].

Non-coding RNAs (ncRNAs) are emerging as key regulators in the development of a variety of disorders, including cancer. With a 200-nucleotide threshold, there are two types of ncRNAs: Long ncRNAs (lncRNAs) are divided into subgroups based on their genomic localization and evolutionary decent, including sense intron RNA, antisense RNA, long intergenic RNA (lincRNA), enhancer RNA, and pseudogenes; Short ncRNAs include miRNAs and siRNAs [36]. The lncRNAs LINC00261 and LINC00665 were elevated after radiotherapy and were found to be a possible negative prognostic sign for overall survival in prostate cancer patients. In long-term survivors, silencing of LINC00665 and LINC00261 reduced survival after re-irradiation and impaired repair of DNA double-strand breaks [37]. Recently, Tang et al., have shown that hypoxia-inhibited mir-133a-3p promotes PCa bone metastasis PI3K/AKT signaling and its low expression was significantly correlated with advanced clinicopathological characteristics [38]. Lekchnov and colleagues examined 84 miRNAs in urine samples. They reported that in the supernatant urine fraction, the most diagnostically significant miRNA pairs were miR-26b.5p, miR-107, and miR-375.3p. In addition, miR-31.5p, miR-200b, miR-16.5p, and miR-660.5p existed in the fraction of extracellular vesicles, which differed between healthy men and BPH patients [39].

Circular RNAs (circRNAs) and lncRNAs have been proposed as a new form of non-coding RNA with regulatory potential in recent years [40]. To investigate circular transcripts found in prostate tumors, ultra-deep RNA-Seq was performed without poly-A selection. In several patient cohorts, circRNAs production was associated with disease progression and an average of 7,232 circRNAs were expressed in each sample. In a loss-of-function screening, it was discovered that 11.3% of circRNAs, which are extremely abundant, are necessary for cell proliferation. For example, circCSNK1G3 stimulates cell growth by interacting with miR-181 [41]. Similarly, Yan et al. discovered 827 upregulated and 1279 downregulated circRNAs by high-throughput sequencing and reported that has-circ-0001165, has-circ-0001085, and has-circ-0004916 were differentially expressed in IFN-γ treated cells by qPCR confirmation [42].

### Proteomics

According to recent cancer studies, transcriptome changes account for only 10 to 20% of proteome changes [43]. Proteomics deals with the identification of proteins and the evaluation of their quantitative properties. Proteomics has been employed in various studies seeking for PCa biomarkers since it can directly reflect cell activity and detect deregulations in the most treatable cellular components [44]. Cell cycle control, DNA repair, proteasomal degradation, and metabolic activity have all been associated with proteomic changes. A study by Shina et al. compared different Omics approaches and evaluated the accuracy of each biomarker. They found that proteomic features were significantly more informative than genomic, epigenomic, or transcriptomic features for predicting biochemical relapse. They also discovered that proteomic subsets of prostate tumors were only weakly linked to their genetic counterparts [45].

Tissue obtained after a biopsy or radical prostatectomy can be utilized for biomarker discovery to and to identify therapy targets based on knowledge of the drug's genome and proteome. Researchers can also explore the molecular basis of cancer growth and progression using in situ histopathology. PPP1CB, UBE2N, and PSMB6 were found to be protein indicators for PCa diagnosis in a study using two-dimensional differential gel electrophoresis-mass spectrometry and Western blot [46]. According to Launonen and colleagues, deletion of the chromatin remodeler SMARCA4 affects chromatin accessibility and expression of a limited selection of AR target genes, as well as proliferation and metastasis of CRPC cells [47].

The proteome of PCa tumor tissue and surrounding tissue is also used for comparative studies to determine the process of carcinogenesis. To rule out interindividual variations, researchers must analyze the same prostate tissue with different histological patterns. Proteins related to smooth muscle contraction, calcium binding, and intercellular interstitial interaction were identified to be increased in the tumor stroma of PCa patients when compared to the neighboring normal stroma [48]. Aiello and coworkers used PCa tissue with a Gleason grade of 6 to look at 132 differentially expressed proteins. Oncogene products, nuclear receptor-supported activators, cytoskeletal proteins, G-protein-coupled receptors, and antiproliferative proteins were all shown to be significantly overexpressed in PCa [49]. In addition, combining proteomics with post-translational adaptations yields more detailed data. In a study by Maria et al. in four prostate cell lines (PC3, DU145, LNCaP, and PNT1A), protein candidates associated with PCa development were identified [50]. The importance of proteomics in the diagnosis and targeted therapy of PCa was reviewed in detail by Tonry et al. [51].

### Epigenomics

Abnormal gene expression is one of the changes that lead to tumor formation. In addition to changes in DNA nucleotide sequence, epigenetic mechanisms can also result in abnormal gene expression. Modification of DNA after transcription and modification of proteins after translation are responsible for epigenetic alteration of DNA structure. Epigenetic changes, unlike gene mutations, are reversible and dynamic. Aberrant DNA methylation, histone modification, chromatin remodeling, and noncoding RNA-mediated abnormal gene expression are all epigenetic processes that have been implicated in the development of PCa [52].

Primary PCa can be distinguished from mCRPC based on the binding of transcription factors and metastasis-specific histone acetylation patterns (H3K27ac) to regulatory elements of the genes *AR*, *HOXB13*, and *FOXA1*, according to a study that integrated the epigenome with genomic and transcriptomic data. Interestingly, during metastatic disease progression, reprogrammed regulatory elements access sites related to prostate organogenesis and hijack latent developmental programs [53]. In addition, there was also a strong association between the inheritance of PCa risk and the density of somatic mutations and prostate-specific regulatory elements. Similarly, in a genome-wide sequencing analysis of histone acetylation, Baca et al. showed that the *FOXA1* cistrome was reprogrammed in the development of neuroendocrine prostate carcinoma (NEPC) after endocrine treatment. *NKX2-1* and *ASCL1* are sufficient to induce de novo H3K27ac at NEPC-enriched *FOXA1* binding sites, leading to NEPC gene expression. Although NEPC is not dependent on *AR*, it maintains expression of *FOXA1* and requires it for gene expression and proliferation that determine neuroendocrine lineage [54].

DNA methylation is a critical component of the epigenetic system. DNA methylation regulates the transcription of the genome, therefore aberrant methylation can cause a variety of diseases, including cancer [55, 56]. Methylation suppresses transcription activity in the promoter region (CpG repeats) by blocking methyl DNA transferases from entering the cell [57]. In over 55% of cases, CpG repeats form clusters whose methylation or demethylation state impedes or activates the transcription process [58]. When malignant tumors, such as PCa, form, these areas are often methylated. In PCa, both hypermethylation and hypomethylation occur, and both methylation status changes contribute to the course of the disease [59, 60]. Hypermethylated genes include *CDH1* and *CD44* are involved in cell adhesion, *PYCARD* is involved in apoptosis regulation, *CDKN2A* is involved in cell cycle regulation, and *GSTP1* and *MGMT* are involved in DNA repair [61]. Anticancer genes such as *RAR*, *RARRES1*, *RASSF1*, and APC are also hypermethylated in PCa [62]. Xu and colleagues discovered a modest increase in overall methylation levels with increasing Gleason score after genome-wide DNA methylation profiling in leukocyte DNA from 280 African American PCa patients. They also discovered 77 differentially methylated regions/genes, including 10 homeobox genes and six zinc finger protein genes, which may be valuable biomarkers for aggressive PCa [63].

Interestingly, studies have shown that the decrease in methylation content in the genome occurs very late in PCa progression and contributes to the heterogeneity of the metastatic tumor. Furthermore, testis antigen genes including *MAGEA1*, *CTAG1B* undergoes CpG island hypomethylation and only expressed in those cell lines that had significant hypomethylation [64]. Similarly, after analysis of CpG methylation signatures in bone metastases and primary PCa by Ylitalo and colleagues, hypomethylation was found to be more common in metastases. In addition, they proposed a methylation classification signature for androgen receptor activity (MCA) that divided metastases into two groups. The MCA-positive metastases had low methylation levels in genes associated with higher AR activity and had a more favorable prognosis after androgen deprivation therapy [65]. However, primary PCa exhibit more pronounced hypomethylation of the LINE1 (Long Interspersed Nuclear Element-1 s) promoter, which can be used for cancer screening, risk assessment, tumor staging, and prognosis [66].

### Metabolomics

In recent years, many metabolomics studies have been performed on PCa samples to characterize the particular metabolic profile associated with PCa progression and to detect metabolic abnormalities that could be used as clinical biomarkers. Mondul et al. studied sera from 74 men with PCa and 74 healthy cases 20 years after blood collection using gas chromatography/mass spectrometry (GC–MS) and liquid chromatography/mass spectrometry (LC–MS). There was a significant negative association between 1stearoylglycerol and PCa risk [67]. They were unable to duplicate the association of 1-stereoylglycerol and thyroxine with higher PCa risk when they performed metabolomics analysis with an additional sample of 200 confirmed PCa cases and 200 controls. Nonetheless, alpha-ketoglutarate and citrate have been associated to an increased risk of aggressive PCa [68]. Positive lipid correlations with overall PCa risk as well as risk for aggressive PCa were also detected in the PLCO cohort study performed on 380 sera from PCa cases and 380 healthy individuals. In contrast to the above studies [67, 68], an association between thyroxine and aggressive PCa was not confirmed. However, the correlation of 2′-deoxyuridine with overall risk of PCa as well as bile acid tauro-beta-muricholate could be repeated [69].

To predict treatment response, Huang and colleagues used LC–MS, to screen 36 PCa patients receiving androgen deprivation therapy (ADT), 18 untreated diagnosed PCa, and 18 healthy controls. Arachidonic acid, pyridinoline, deoxycholic acid, tryptophan, and the nucleotide deoxycytidine triphosphate were discovered as possible biomarkers for predicting ADT response. All of these markers were altered in PCa patients compared with healthy controls. In contrast, serum levels returned to those of the control group in subjects who responded to ADT. This suggests that serum levels of these metabolites could be used to predict response to endocrine therapy [70]. Using high-performance liquid chromatography and mass spectrometry (HPLC–MS), Andras and colleagues analyzed the sera of 90 individuals with PCa and benign prostatic hyperplasia (BPH). From the data set, a discovery set (n = 59) and a validation set (n = 31) were created. In the training set, the resulting score, which comprised markers including homocysteine inosine, methyladenosine, lipoic acid, hydroxymelatonin, and decanoilcarnitine, had a sensitivity of 74% and a specificity of 76%. In the validation set, the score distinguished PCa from BPH with a sensitivity of 88% and a specificity of 60% [71].

Many noninvasive assessments have been used to predict PCa risk. Urinary changes linked to PCa risk were described by Kosti et al. [72]. They employed LC–MS to evaluate urine concentrations of 15 estrogen metabolites in 77 incident PCa cases, 77 healthy controls, and 37 subjects lacking PCa evidence based on prostate biopsy. When compared to healthy controls, PCa patients had considerably lower levels of 16-ketoestradiol (16-KE2) and 17-epiestriol (17-epiE3). Roberts et al. used nuclear magnetic resonance spectroscopy to analyze seminal plasma prior to or at least one month after prostate biopsy. The presence of a primary Gleason pattern 4 (present versus absent) in these samples was associated with higher levels of lipids/lipoproteins, lactate, and pyruvate and lower levels of citrate, spermine, and myoinositol [73]. For a comprehensive analysis of studies performed on metabolomics and PCa, see review by Kdadra et al. [74]. Cerrato and colleagues discovered that amino acids and carnitine derivatives were associated with PCa by comparing urine samples from BPH and PCa. Although previous research points to their importance in cancer biomarker discovery, these families of chemicals are often overlooked in conventional metabolomics testing [75].

## Single-cell omics

Advances in DNA sequencing technology have enabled more thorough investigation of genomic features in treatment-resistant tumors, although the associated analytical tools are only now beginning to demonstrate their value. For most omics, single-cell (SC) omics is theoretically possible. In many different cell types, SC analysis reveals DNA mutations and altered gene expression and can identify resistant cells before and after treatment. It can also quantify heterogeneity within and between tumors, characterize mutation rates, and identify unusual cell types, ultimately helping to develop diagnostic and treatment guidelines [76]. Researchers could also use SC omics to analyze tumor microenvironment (TME) function in cancers that are resistant to immune treatment, such as PCa, which has a high degree of clinical heterogeneity and clonal genetic diversity [77]. The first level of gene control is DNA accessibility, and transcriptome changes are increasingly used to find molecular predictors of response to cancer treatment.

At PCa, non-genetic alterations in the transcriptome, chromatin structure, and DNA accessibility of transcription factor binding motifs are increasingly common but less well understood. Taavitsainen et al. have taken a molecular look at the emergence of resistance to *AR* -targeted treatments. They show that chromatin remodeling and gene expression changes in single cell populations are accompanied by several PCa-associated transcription factors such as *MYC*, *HOXB13*, and *GATA2*, which are outnumbered in several cell clusters. Moreover, patient responses to treatment can be stratified using transcriptional patterns specific to persisted cells [78]. More recently, Anti-programmed cell death protein 1 (*PD-1*) and YY001 (a novel EP4 antagonist) have been proposed to have anti-cancer activity in vitro and in vivo studies on clinical samples. EP4 regulates the TME of PCa patients by expression in epithelial cells and various immune cells. The development, maturation, and immunosuppressive function of myeloid-derived suppressor cells (MDSCs) were hindered by YY001, while the proliferation of T cells and their ability to fight cancer were increased. When combined with anti PD-1 antibodies, YY001 transforms non-responsive PCa into responsive tumors, resulting in significant tumor shrinkage, long-term survival and enhanced immunological memory [79].

To decipher the cellular architecture of neuroendocrine differentiation in human PCa, the transcriptome of 21,292 cells from 6 CRPC was sequenced. It became clear that neuroendocrine tumor cells have a luminal-like epithelial phenotype and are derived from luminal-like malignant cells rather than the basal compartment. These results were validated by microarray analyzes, and gene signatures associated with neuroendocrine differentiation were also clarified [80]. Song et al. identified club cells in radical prostatectomy specimens and localized PCa biopsies by diagnosing highly expressed genes such *MMP7*, *PIGR*, *LTF*, and *CP*, as well as the significantly reduced expression of *LCN2* and *SCGB3A1*. These club cells are more sensitive to androgens, which may predispose to tumor cell transformation and promote prostate tumorigenesis [81]. Similarly, Chen et al. found that ectopic expression of KLK3 was connected with micrometastases in a transcriptome analysis of 36,424 single cells from 13 prostate carcinomas. In addition, there is close cell–cell communication between cells and activated endothelial cells are enriched in CRPC cells and promote cancer cell invasion [82].

## Multi omics

**Fig. 2:**
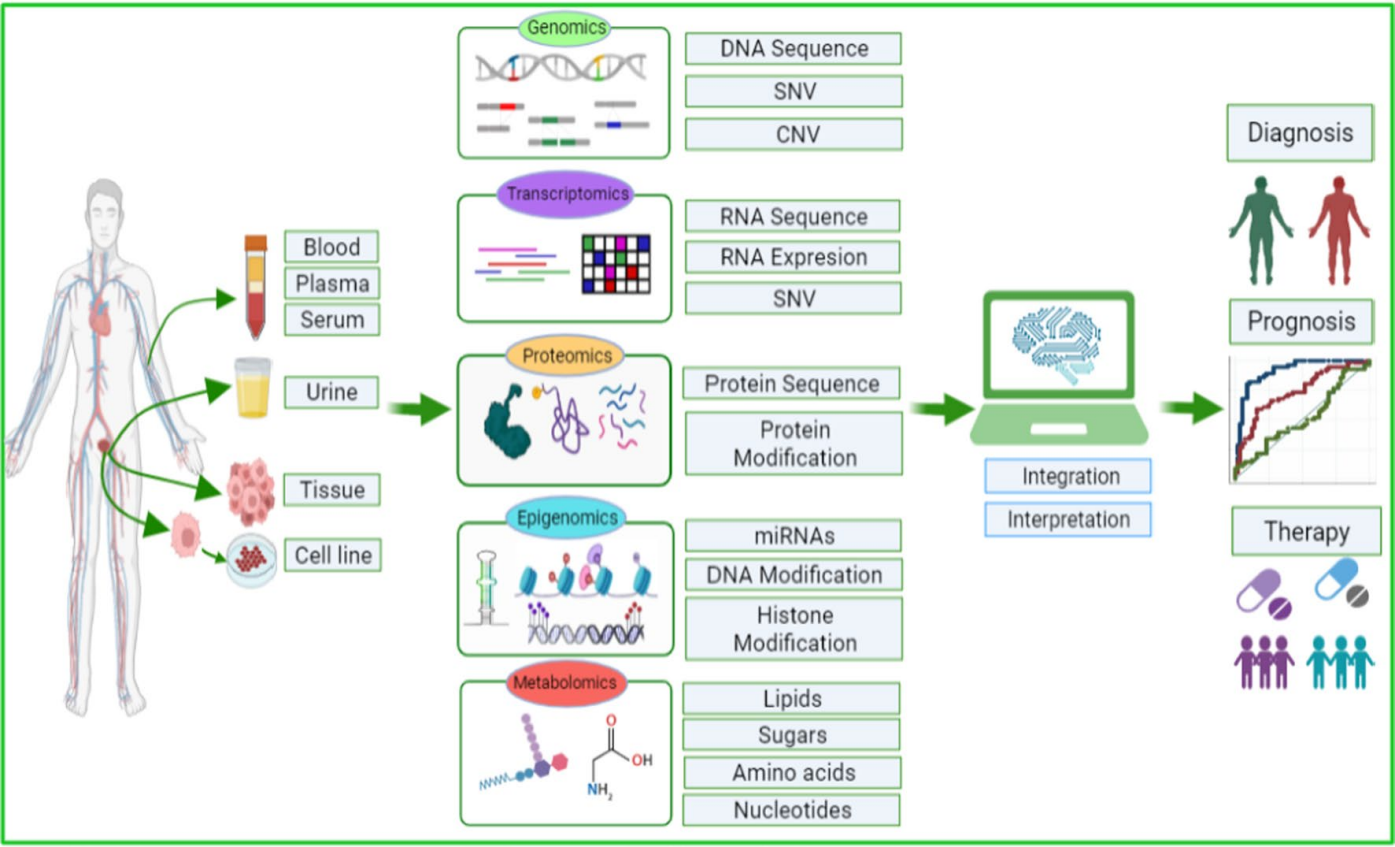
Multi-omic strategy in Prostate cancer study. Omics investigations discover molecular characteristics of the PCa. Integration of multiple omics provide a more comprehensive view of the PCa, leading to cancer detection, screening, patient classification, and assessment of treatment response. *SNV* single-nucleotide variation, *CNV* copy number variation

PCa is associated with multisystemic and multilayered pathogenic changes during its development and progression. Omics studies at one level often reach their limits. In contrast, combining multiple Omics data to develop targeted indicators for PCa therapy may be more effective and thorough. Therefore, integrating data generated at each level is essential to understand the complex nature of cancer and get a holistic overview of genomic instability events, which otherwise is not possible by single Omics data analysis [83]. In the recent decade, several clinical and preclinical studies showed the importance of data integration to get a clear and concise picture of the disease under investigation. The PCa multi-Omic combination study strategy is depicted in Fig. 2.

PCa is correlated with molecular abnormalities, as shown by integration of metabolomics and transcriptomics. According to this study, the metabolite sphingosine has high specificity and sensitivity for distinguishing prostate cancer from benign prostatic hyperplasia. Loss of the tumor suppressor gene sphingosine-1-phosphate receptor 2, which is downstream of sphingosine, suggests a potentially important oncogenic pathway that can be therapeutically targeted [20]. Ren et al., by a genome-wide sequencing and transcriptomic study on samples from 65 untreated PCa patients, found that the high frequency of *CHD1* deletion in Chinese patients was associated with a higher proportion of mutations in the androgen receptor upstream activator gene and a low *TMPRSS2*-*ERG* fusion rate. They also identified *PCDH9* as a critical tumor suppressor gene because it is missing in 23% of cancers. Besides, PLXNA1 gene was amplified in approximately 17 percent of tumors in a group of PCa patients from multiple institutions, which was confirmed by functional and clinical analyses. *PLXNA1* overexpression was found to accelerate prostate tumor development and to predict metastasis, biochemical recurrence, and poor survival rates independently [84].

Several research papers have recently demonstrated that merging omics datasets leads to a better knowledge and picture of the system under study. Kwon et al. discovered 70 mutant peptides in PCa cell lines. After parallel reaction screening, they found that the levels of seven mutant peptides changed in PCa, with *CAPN2* D22E being the most dramatically increased. They concluded that modified mutant peptides in PCa could be exploited to generate novel biomarkers for advanced PCa [85]. Semen is an ideal sample for biomarker discovery because it is close to the prostate and can be analyzed noninvasively. Using data from histology, transcriptomics, seminal fluid proteomics, tissue specificity, androgen regulation, and cell line secretion omics, Drabovich et al. found 147 potential markers. Prostate-specific and androgen-regulating protein TGM4 has been shown to be more effective than age and blood PSA levels in predicting PCa by biopsy. TGM4 was increased 3.7-fold and the AUC was 0.66 in the independent validation data set for PCa patients with a serum PSA level of 4 ng/ml and age of 50 years [86].

Due to the vast range of molecular characteristics of PCa, identification of metabolic alteration is an important step in obtaining a more accurate diagnosis and prognosis. Gao et al. discovered significant changes in cell phenotype between the LASCPC-01 and LNCAP cell lines based on the transcriptomic and metabolomic integration. Upregulation of 62 genes in LASCPC-01 and 112 genes in LNCAP was discovered through enrichment analysis of transcriptome data. Chemical enrichment analysis of metabolome and liposome Omics revealed 25 significantly distinct metabolite groups. LNCAP had increased one-carbon metabolism as a glycolytic intermediate pathway and reduced levels of the mitochondrial lipid transporter carnitine, whereas LASCPC-01 had more glycolytic activity and lower triglyceride levels [87].

Multiple cancer-related pathways can be inhibited simultaneously with drugs, increasing treatment options and decreasing drug resistance. A team of researchers processed DNA sequencing, gene copy number, DNA methylation, and RNA-Seq data from cancer patients to create a network of driver signaling pathways using a combined analysis algorithm [88]. Drake et al. used phosphoproteomics, transcriptomics, and genomics to identify the biological mechanisms that impede response to antiandrogenic therapy in patients with mCRPC. According to their analysis, a number of signaling proteins found in these pathways, including *PTK2*, *PRKAA2*, *RPS6KA4*, *PRKDC* and members of the *CDK* family, may serve as novel therapeutic targets or biomarkers for prostate cancer [89]. In addition, the combination of multi-Omic and network pharmacology is becoming increasingly common [90]. A network of putative PCa targets and active chemicals extracted from Hedyotis diffusa Willd was constructed. Quercetin and Ursolic acid were found to be the major components involved in the treatment of PCa [88] (Table 1).

**Table 1:** Characteristics of remarkable cancer databases

Study	Sample	Omics data	Comparison	Major findings
Latonen et al. [128]	Tissue	P + T	PCa vs. CRPC	There are several miRNA target correlations at the protein level but none at the mRNA level. They discovered two metabolic changes TCA cycle during the expansion and advancement of PCa
Yan et al. [129]	Tissue	L + M + T	SPOP-wild vs SPOP-mutated	All SPOP mutations were found in the MATH domain. Three metabolic pathways, including fatty acid metabolism, TCA cycle, and glycerophospholipid metabolism, were upregulated in SPOP mutant tissues
Oberhuber et al. [130]	Tissue	M + P + T	STAT3 low vs STAT3 high	At the transcriptome level, OXPHOS is upregulated in PCa, as is the TCA cycle/OXPHOS at the proteome level. A promising independent prognostic marker in PCa is PDK4, a critical regulator of the TCA cycle
Murphy et al. [131]	Tissue serum	E + M + P + T	BPH vs. PCa	Higher accuracy in predicting PCa aggressiveness compared to clinical features alone or individual omics data with Ordinal C‐Index value of 0.94 and Multi AUC value of 0.91
Itkonen et al. [132]	Cell line	M + P + T	CDK9 inhibitor treated vs. untreated	Inhibition of CDK9 causes acute metabolic stress in prostate cancer cells by consuming ATP and triggering rapid and sustained phosphorylation of AMPK, as well as dramatically downregulating oxidative phosphorylation in mitochondria and accumulation of acylcarnitines, metabolic intermediates in fatty acid oxidation
Kamoun et al. [133]	Tissue	E + G + T	PCa vs. NAT	A group of 36 transcriptomic biomarkers outperformed the most commonly used prognostic molecular signatures in identifying a subpopulation of patients without biochemical relapse
Gómez et al. [134]	Urine serum	M + T	Low vs. high grade PCa	Between the two groups of patients, there were significant changes in 36 metabolic pathways, including glycine, glucose, and 1-methlynicotinamide, metabolites important for energy metabolism and nucleotide synthesis
Paez et al. [135]	Tissue	P + T	PRAD vs. NAT	HO-1 is related with cellular cytoskeleton integrity, and its stimulation in PCa cells resulted in reduced cell trajectory and velocity, a lower frequency of migratory events, and a markedly increased proportion of filopodia-like protrusions that facilitate attachment between adjacent cells
Sial et al. [136]	In silico	E + P + T	Data from HPA, CTD, GEO, and TCGA	TMED2 appears to play a key role in the formation and progression of PCa, as its expression was found to be higher in PCa patients than in healthy controls, and it was also linked to relapse-free and overall survival
Wang et al. [137]	In silico	E + T	Data from TGCA	In silico analysis of Omics data from the cancer database showed that a combination of TELO2, JMJD6, miR-378a, miR-143, MED4, and ZMYND19 had a five-year relapse predictive power of AUC = 0.789
Kiebish et al. [138]	Serum	L + M + P	BCR vs. non-BCR	TNC, Apo-AIV, 1- MA, and PA had a cumulative predictive power of AUC = 0.78, which increased sensitivity (AUC = 0.89) when paired with PCa stage and Gleason score

PCa: Prostate cancer, BCR: Biochemical Recurrence. CRPC: Castration-resistant prostate cancer, TCA: the tricarboxylic acid, OXPHOS: oxidative phosphorylation, BPH: Benign prostatic hyperplasia, AUC: area under the curve, NAT: Normal adjacent tissue, BCR: Biochemical recurrence, PRAD: Prostate Adenocarcinoma, CTD: The Comparative Toxicogenomics Database, GEO: Gene Expression Omnibus, HPA: The Human Protein Atlas, TCGA: The Cancer Genome Atlas, G: Genomics, E: Epigenomics, M: Metabolomics, P: Proteomics, T: Transcriptomics

## Computational algorithms for data integration

Since the introduction of high-throughput technologies such as next-generation sequencing (NGS) and microarrays the amount of biological data available has increased dramatically, throwing biology into the era of Big Data [91]. Conventional data analysis tools have struggled to extract biological insights about various features of genes, proteins, or biomolecules from these high-dimensional datasets in a timely and cost-effective manner [92]. The physiological and computational complexity of multi-omic datasets, as well as noise ratios and lack of statistical power, have further complicated the interpretation of data in cancer research [93]. To gain new insights and expand our understanding of cancer, improve diagnostics, and develop individualized treatments, biomedicine requires advanced informatics tools. There are numerous attempts in computational biology to develop new algorithm to integrate datasets generated by omics techniques.

Drake et al. combined several datasets of mutations, transcriptional alterations, and phosphoproteome activities using the TieDIE algorithm, which provide an integrative, pathway-based reference for drug prioritization in individual patients by shedding light on the diversity of activated signaling pathways in metastatic CRPC [89]. Sinha et al. conducted one of the intriguing studies combining omic methods on 76 PCa specimens [94]. They employed supervised machine learning to train and evaluate CNV, methylation, RNA, and protein biomarkers after profiling the genome, transcriptome, proteome, and epigenome of intermediate-risk PCa patients. They discovered biomarkers using matched biomolecule combinations and found that predictive biomarkers that combined genomic or epigenomic with proteomic features significantly outperformed biomarkers based on a single data type.

These studies have demonstrated the value of integrating multi-omic data compared to single-omics analyzes. The use of a multi-omic approach has led to the development of a variety of tools, methods, and platforms for the analysis, visualization, and interpretation of multi-omic data. The Integrated Heterogeneous Multi-Omic Data Analysis (iODA) tool is a system-level graphical user interface that uses common pathways from single Omics datasets such as mRNA, miRNA, and protein-DNA interaction (ChIP-Seq) data. The pathways shared by multiple Omics data have been evaluated as a key component of iODA's systematic description of disease progression, which is critical for the in-depth study of complicated pathogenic mechanisms [95]. Recently, Saghaleyni and colleagues developed a module with eight machine learning algorithms for analyzing secreted protein profiles using gene expression data from thousands of normal human tissue and tumor samples. *KIF20A* and *KIF23*, members of the kinesin family, were consistently among the top genes linked to malignant transformation [96]. Multi-Omic Graph Convolutional NETworks (MOGONET) effectively categorize multi-omic data, by both omics-specific learning and cross-omics correlation learning. Using mRNA expression data, microRNA expression data, and DNA methylation data, it identifies important biomarkers in various cancers [97]. Kaur et al. published a comprehensive review of CAs that can handle multi-Omic data [98].

Large collections of genomic datasets from diverse samples, such as biopsies, cell lines, and images, have been stored in public databases, which has improved our understanding of the genomic heterogeneity of cancer cells and supported the identification of novel patient-specific treatments. The Gene Expression Omnibus (GEO) and ArrayExpress are high-throughput gene expression databases that also includes hybridization arrays, and microarrays in various diseases [93]. Similarly, The Cancer Genome Atlas (TCGA) is a groundbreaking cancer genome project that has scrutinized more than 20,000 samples of primary and benign cancers from 33 cancer types for molecular signatures [99]. These data have increased our ability to identify, treat, and prevent cancer, and they will be available to the scientific community. Using TGCA, transcriptomics and survival data from 498 patients with PCa were analyzed to find potential biomarkers for prognosis. *TMED10*, *TMED2*, and *SEC31A* were found to be positive prognostic biomarkers for PCa because their high expression was associated with better overall survival in PCa patients [100]. Gao et al. discovered MiR-452-5p is downregulated in prostate cancer by combining data from GEO, ArrayExpress, and the TCGA database on 1007 prostate cancer samples and 387 noncancerous samples [101]. Following bioinformatic analysis revealed that the genes targeted by MiR-452-5p are thought to be involved in biological processes such as Ras signaling, signaling pathways regulating stem cell pluripotency, and transforming growth factor beta signaling. Table 2 contains a list of valuable cancer-related databases.

**Table 2:** several of the ground-breaking PCa multi-omics research

Name	Data	Main features	Link
TCGA [99]	over 20,000 primary cancer and matched normal samples spanning 33 cancer types	2.5 petabytes of genomic, transcriptomic, epigenomic, and proteomic data have already improved our ability to diagnose, treat, and prevent malignancies	https://portal.gdc.cancer.gov
GEO [139]	Stores curated datasets and original submitter-supplied records	A global public repository for microarray, next-generation sequencing, and other types of high-throughput functional genomics data provided by the scientific community	https://www.ncbi.nlm.nih.gov/geo
ArrayExpress [140]	Public repository of curated gene expression profiles by microarray data	Gene expression profiles can be retrieved using gene names and attributes such as Gene Ontology terminology, and gene expression profiles can be displayed	www.ebi.ac.uk/arrayexpress
ICGC [141]	Projects, including ICGC, TCGA, Johns Hopkins University, and the Tumor Sequencing Project, covering somatic mutations, CNV, structural rearrangements, gene expression, microRNAs, and DNA methylation data	Uses a web-based graphical user interface to provide researchers with a variety of options for searching and analyzing data, and to help them create complex searches across multiple data sets	http://dcc.icgc.org
MMHCdb [142]	Data is gathered from direct researcher submissions and a number of bioinformatics tools connected to cancer research databases	a comprehensive, well curated collection of human cancer mouse models	http://tumor.informatics.jax.org/mtbwi
PIXdb [33]	gene expression data from ArrayExpress, GEO, TCGA, and ICGC	Perform exploratory and in-depth analyses on these datasets, either individually or in combination, with the ability to track molecular events across different stages of prostate cancer development and progression	https://pixdb.org.uk
ONCOMINE [143]	Over 4700 microarray experiments yielded 65 gene expression data sets with nearly 48 million gene expression data	Clinical and pathological assessments and differential expression analyses comparing a range of cancer subtypes	https://www.oncomine.com
cBioPortal [144]	Web resource to easily understand multidimensional cancer data such as genetic, epigenetic, gene expression and proteomic data through exploration, visualization and analysis	Multidimensional cancer genomics data can be explored, visualized, and analyzed using this web resource	https://www.cbioportal.org/
CancerSEA [145]	Data from 41,900 cancer single cells from 25 cancer types	At the single-cell level, investigate the many functional states of cancer cells	http://biocc.hrbmu.edu.cn/CancerSEA
COSMIC [146]	Largest somatic mutation library, including over 6 million coding alterations in 1.4 million tumor samples collected from over 26,000 studies	Largest somatic mutation database; genome sequencing paper curation	http://cancer.sanger.ac.uk
canEvolve [147]	Data from 90 studies involving more than 10,000 patients	Comprehensive analysis of tumor profile;	www.canevolve.org
SomamiR [148]	Experimentally validated miRNA target sites with 388 247 somatic mutations	Correlation between somatic mutation and microRNA; genome-wide displaying	http://compbio.uthsc.edu/SomamiR
CCLE [149]	Genomics and transcriptomics data of 60 cancer cell lines	Validation of cancer targets and therapeutic efficacy in commonly studied cancer cell lines. To translate cell line genomics and transcriptomics into cancer patient stratification	https://sites.broadinstitute.org/ccle/
MethyCancer [150]	Relationship among DNA methylation, gene expression and cancer	To investigate the relationship between gene expression, DNA methylation and carcinogenesis. It contains both high-integrity DNA methylation, cancer-related gene, mutation, and cancer data from public sources	http://methycancer.psych.ac.cn
NONCODE [151]	Annotation of 167,150 lncRNA in human diseases	ncRNAs; lncRNAs; up-to-date and comprehensive resource	http://www.noncode.org/

## Challenges

Although it is the most reliable method, assessment of tumor stage and biology using biopsies remains challenging and tedious and carries significant potential side effects and complications. In addition, the inherent risk of missing more advanced/aggressive tumor areas and the high variability between observers lead to misclassification of samples [102, 103]. More importantly, these classifications for clinical decision making do not take into account the different tumor phenotypes and therefore do not reliably predict individual patient risk [104]. Furthermore, clinicians and patients often opt for intensive therapy based on findings and psychological distress without clear evidence of aggressive disease, which contributes significantly to extensive overtreatment [105, 106]. Current estimates suggest that up to 50% of PCa cases eventually receive intensive treatment, while only 20% have aggressive cancer. After intensive therapy, patients who undergo surgery or radiation therapy experience severe side effects, with up to 50% of patients relapsing.

Although significant progress has been made in recent years in identifying potential biomarkers, there are still gaps between discovery research and clinical application [107]. Biomarkers for PCa have been proposed, including branched-chain amino acids and citrate [108–110], which have been reported for other diseases [111], implying that the biomarkers found are likely not specific to prostate cancer. Another crucial concern is whether the alterations in plasma metabolites are reflective of tumor cell changes or not. To improve the predictive power of potential diagnostic biomarkers, experiments must be carefully designed. For example, sample size should be determined according to the type of sample, technical characteristics of omics, and statistical analysis methods. In addition, standardization of sample collection and storage may help reduce biological variability between studies [112, 113]. Follow-up studies are also needed to track survival rates and determine whether certain screening methods can reduce mortality [114]. Finally, more thorough validation is needed before results can be used in routine clinical care. Fortunately, the less invasive and rapid liquid biopsy procedure meets the need for large screens in healthy individuals. However, current liquid biopsy tests lack accuracy and consistency [115]. Standardization of liquid biopsy testing procedures and analysis platforms will be necessary in the future so that results from different studies can be compared and combined [116].

Integrating biomarkers across multiple omics systems dramatically improves predictive accuracy. Omics enables researchers to organize, monitor, compare, and assess patterns of molecular changes such as DNA mutations and CNV, protein modifications, mRNAs and miRNAs expression patterns, and epigenetic changes in individual patients to uncover the molecular signatures underlying complex cellular phenotypes [117]. The introduction of technologies to capture and integrate information from these large, multidimensional data sets will improve the translation of omics data into clinical practice. Luckily, CAs for the analysis of multi-omic data, such as machine learning techniques, have been proposed that enable the identification of multi-omic signatures associated with disease phenotypes [118]. These many molecular profiles will provide a comprehensive picture of PCa screening and diagnosis and accelerate the search for candidate biomarkers. There are two approaches to data integration. The first strategy relies primarily on prior knowledge of known cancer pathways and processes. However, linking separate molecular data sets between databases is challenging. For example, regulatory data from ENCODE are not necessarily linked to specific genes that can subsequently be mapped to KEGG pathways. Similarly, metabolite data are weakly linked in current versions of gene-centric pathway networks. The second approach neglected existing knowledge of metabolic pathways and network interactions in cells and tissues and prioritized finding queries that change in a coordinated manner [119].

While the use of omics data, particularly genomics, has led to insights that have been used in clinical oncology to support treatment decisions, SC analysis, which has recently emerged as a promising approach for capturing invaluable cellular-level data, such as TME and metastasis status [75, 120]. However, it is limited by artifacts caused by evolutionary dynamics during the growth of a laboratory clone and by the fact that it can only examine a small number of founder cells [121]. Similarly, since proteins, lipids, glycans, and metabolites cannot be replicated like nucleic acids, single cell omics would not be able to effectively analyze proteomics, lipidomics, glycomics, or metabolomics.

Because most RNA sequencing data are from the bulk of malignancies, they cannot account for PC heterogeneity. This is because the transcriptome is the consequence of numerous biological processes that contribute to differential gene regulation, and these activities are not always synchronized in the tumor bulk [122, 123]. Cancer stem cells and circulating tumor cells are among the rare tumor cells that can be effectively detected by the SC method. It is interesting that topographic SC sequencing can detect tumor cell invasion and metastasis [124]. In addition, SC multiple sequencing examines DNA methylation and the state of chromatin in the SC to define intratumoural heterogeneity, which in turn leads to tailored therapeutic approaches [125]. Moreover, coupling SC sequencing with other technologies, such as CRISPR screening, enables the functional study of heterogeneous cell populations and facilitates the study of the interplay between genes and regulatory elements. A significant cost reduction is also foreseen [126]. Recently, the development of novel Nano-microarrays has made it possible to process thousands of single cells in parallel, which, combined with dynamic secondary ion mass spectrometry that has three dimensional scanning capacity and higher resolution, could greatly improve the sensitivity of single-molecule quantification for all classes of biomolecules [127].

## Conclusion

Overall, this work highlights the need for the use of multi omic approach to achieve better outcomes in the treatment of PCa patients. We are just beginning to collect complete, unbiased multi-omic data to develop the appropriate statistical and annotation tools to help us understand these complicated data sets and extract biologically and clinically relevant information. Because multi-omic data are expensive and time-consuming, access to appropriate tissue samples and biopsy material is essential for generating multi-omic data. As described in this review, various molecular characteristics of tumor cells revealed by the multi-omic approach lead to effective screening methods for early cancer detection, screening, patient selection strategies, or assessment of response to treatment. In the future, more advanced and innovative approaches to the integration and interpretation of multiple omics data should be developed.

## Data Availability

All the data generated and analyzed during this study are included in the manuscript and the additional materials.

## Abbreviations

APC: APC regulator of WNT signaling pathway
AR: Androgen receptor
CAPN2: Calpain 2
CDH1: Cadherin 1
CDKN2A: Cyclin dependent kinase inhibitor 2A
CENPA: Centromere protein A
CHD1: Chromodomain helicase DNA binding protein 1
CP: Ceruloplasmin
DDC: Dopa decarboxylase
ERG: ETS transcription factor ERG
EZH2: Enhancer of zeste 2 polycomb repressive complex 2 subunit
FOXA1: Forkhead box A1
GABPB1: GA binding protein transcription factor subunit beta 1
GATA2: GATA binding protein 2
GSTP1: Glutathione S-transferase pi 1
HEATR5B: HEAT repeat containing 5B
HOXB13: Homeobox B13
JMJD6: Jumonji domain containing 6, arginine demethylase and lysine hydroxylase
KIF20A: Kinesin family member 20A
KIF23: Kinesin family member 23
KLK3: Kallikrein related peptidase 3
LCN2: Lipocalin 2
LTF: Lactotransferrin
MED4: Mediator complex subunit 4
MGMT: O-6-methylguanine-DNA methyltransferase
MMP7: Matrix metallopeptidase 7
MYC: MYC proto-oncogene, bHLH transcription factor
PCDH9: Protocadherin 9
PIGR: Polymeric immunoglobulin receptor
PIK3CB: Phosphatidylinositol-4, 5-bisphosphate 3-kinase catalytic subunit beta
PLXNA1: Plexin A1
PPP1CB: Protein phosphatase 1 catalytic subunit beta
PSMB6: Proteasome 20S subunit beta 6
PYCARD: PYD and CARD domain containing
RARRES1: Retinoic acid receptor responder 1
RASSF1: Ras association domain family member 1
RB1: RB transcriptional corepressor 1
RFX6: Regulatory factor X6
SCGB3A1: Secretoglobin family 3A member 1
SEC31A: SEC31 homolog A, COPII coat complex component
SMARCA4: SWI/SNF related, matrix associated, actin dependent regulator of chromatin, subfamily a, member 4
TELO2: Telomere maintenance 2
TGM4: Transglutaminase 4
TMED2: Transmembrane p24 trafficking protein 2
TMED10: Transmembrane p24 trafficking protein 10
TMPRSS2: Transmembrane serine protease 2
TNC: Tenascin C
TP53: Tumor protein p53
TP63: Tumor protein p63
UBE2N: Ubiquitin conjugating enzyme E2 N
ZMYND19: Zinc finger MYND-type containing 19
ZNF292: Zinc finger protein 292
SPOP: Speckle-type POZ protein
MATH: Meprin and TRAF homology
CDK9: Cyclin-dependent kinase 9
AMPK: AMP-activated protein kinase
HO-1: Heme oxygenase 1
1-MA: 1-Methyladenosine phosphatidic acid
Apo-AIV: Apolipoprotein A1V
PDK4: Pyruvate dehydrogenase kinase 4
